# Sulforaphane metabolites reduce resistance to paclitaxel via microtubule disruption

**DOI:** 10.1038/s41419-018-1174-9

**Published:** 2018-11-14

**Authors:** Yalin Wang, Yan Zhou, Zhongnan Zheng, Juntao Li, Yuting Yan, Wei Wu

**Affiliations:** 1 Department of Biochemistry and Molecular Biology, School of Basic Medical Sciences, Capital Medical University, Beijing, P.R. China; 2 Beijing Key Laboratory of Tumor Invasion and Metastasis Research, Capital Medical University, Beijing, P.R. China; 3 Institute of Brain Tumor, Beijing Institute for Brain Disorders, Capital Medical University, No. 10, Xitoutiao, You An Men Wai Ave., Feng Tai District, Beijing, 100069 P.R. China

**Keywords:** Cell biology, Molecular biology

## Abstract

Long treatment with paclitaxel (PTX) might increase resistance and side-effects causing a failure in cancer chemotherapy. Here we uncovered that either sulforaphane-cysteine (SFN-Cys) or sulforaphane-N-acetyl-cysteine (SFN-NAC) induced apoptosis via phosphorylated ERK1/2-mediated upregulation of 26 S proteasome and Hsp70, and downregulation of βIII-tubulin, XIAP, Tau, Stathmin1 and α-tubulin causing microtubule disruption in human PTX-resistant non-small cell lung cancer (NSCLC) cells. Knockdown of either βIII-tubulin or α-tubulin via siRNA increased cell sensitivity to PTX, indicating that these two proteins help cells increase the resistance. Tissue microarray analysis showed that overexpression of βIII-tubulin correlated to NSCLC malignant grading. Immunofluorescence staining also showed that SFN metabolites induced a nest-like microtubule protein distribution with aggregation and disruption. Co-immunoprecipitation showed that SFN metabolites reduced the interaction between βIII-tubulin and Tau, and that between α-tubulin and XIAP. The combination of PTX with SFN metabolites decreased the resistance to PTX, and doses of both PTX and SFN metabolites, and enhanced apoptosis resulting from activated Caspase-3-caused microtubule degradation. Importantly, the effective dose of SFN metabolites combined with 20 nM PTX will be low to 4 μM. Thus, we might combine SFN metabolites with PTX for preclinical trial. Normally, more than 20 μM SFN metabolites only leading to apoptosis for SFN metabolites hindered their applications. These findings will help us develop a low-resistance and high-efficiency chemotherapy via PTX/SFN metabolites combination.

## Introduction

Paclitaxel (PTX) was used to treat a variety of cancers efficiently by interfering with microtubule dynamics^1^. However, recent reports showed that PTX therapy increased cell resistance and promoted metastasis^2^. The combination of drugs was proved to lower drug resistance, doses and toxicity to reach synergistic effects of anti-cancer^3^. Sulforaphane (SFN) extracted from cruciferous vegetables was a very powerful agent to inhibit a number of cancers. SFN combined with PTX was demonstrated to promote PTX-induced apoptosis^4,5^. SFN was metabolized in vivo to produce sulforaphane-cysteine (SFN-Cys) and sulforaphane-N-acetyl-cysteine (SFN-NAC), which were more abundant in lungs and plasma compared with SFN^6^. We previously demonstrated that SFN metabolites inhibited cancer proliferation and autophagy^7^, and induced apoptosis via microtubule disruption^8^. Unluckily, the working dose is more than 20 μM for each so that these potential drugs could not be applied for patients so far. Interestingly, similar to PTX, SFN metabolites also have potential to disturb microtubule dynamics, thus the combination of PTX with SFN metabolites might lower their working doses, cell toxicity and resistance, and elevate anti-cancer efficacy via regulating microtubules and microtubule associated proteins leading to the intrinsic cleaved-Caspase-3-mediated apoptosis.

The drugs that either stabilize or destabilize microtubules have potential to bind to soluble or insoluble α-tubulin to induce microtubule dysfunction and apoptosis^9^. PTX binds to β-tubulin^10^, while SFN binds to α-tubulin^11^, and these two tubulins are the targets of anti-cancer drugs. Studies showed that overexpression of microtubule associated proteins class III beta-tubulin (βIII-tubulin), anti-apoptotic protein X-linked inhibitor of apoptosis protein (XIAP), microtubule stabilizing protein Tau, microtubule destabilizing protein Stathmin1 and Hsp70 was considered to be the main reason producing resistance. Increased expression of βIII-tubulin promoted cell survival and drug resistance to PTX in NSCLC cells^12,13^. XIAP mainly functions as a potent suppressor via blocking Caspase-3-mediated apoptosis^14^. Increased XIAP was shown to correlate with resistance of cancer cells to drugs and radiotherapy^15^, whereas decreased XIAP sensitized cancer cells to apoptosis^16^. Tau promotes tubulin assembly and microtubule stabilization, and may bind to the PTX-binding site on the inner surface of the microtubule^17^. High expression of Tau was found to be supportive to the chemo-resistance to PTX, while patients with low expression of Tau could be sensitive to PTX therapy^18,19^. Stathmin1, also known as oncoprotein 18, is a cytosolic phosphoprotein and a key regulator of cell division due to its microtubule depolymerization. High Stathmin1 level is associated with chemo-resistance and poor prognosis in gastric cancer patients^20^. Besides, studies showed that elevated expression of Hsp70 in cancer cells may be responsible for tumor progression by providing resistance to chemotherapy, and knockdown of Hsp70 induced remarkably sensitivity to PTX -induced apoptosis^21^.

We previously demonstrated that SFN metabolites induced α-tubulin degradation and microtubule disruption via ERK1/2 phosphorylation^8^, and SFN-mediated upregulation of 26S proteasome via sustained ERK1/2 phosphorylation leading to microtubule disruption and cell apoptosis^22^. Proteasome-mediated degradation regulates numerous cellular proteins to maintain normal functions of cells^23^. Studies showed that degradation of both α-tubulin and β-tubulin in a variety of human cancer cells could be proteasome-dependent and be induced by SFN^24^. The level of XIAP was regulated depending on activation of the 26S proteasome^25^. Hsp70 and Stathmin1 could be cleaved dependent of ubiquitination and degradation by 26S proteasome; misfolded and aggregated Tau can be degraded by enhancement of proteasomal activity in neurons^23,26–28^. Just recently, we found that SFN metabolites disrupted microtubules and incredibly induced apoptosis via ERK1/2 phosphorylation, downregulation of α-tubulin, microtubule associated proteins, such as Stathmin1, etc. in a couple of cancer models^8^. Therefore, the downregulation of α-tubulin, βIII-tubulin and XIAP, degradation of Tau and Stathmin1 by SFN metabolites might induce microtubule disruption.

Taken together, we hypothesized that SFN metabolites might regulate the levels of βIII-tubulin, XIAP, Tau, Hsp70 and Stathmin1 protein by activated ERK1/2-mediated 26S proteasome, thereby interfering with dynamics of microtubules, reducing the resistance to PTX and synergistically promoting apoptosis in human PTX-resistant NSCLC cells. Therefore, investigation of the underlying mechanisms which combination of PTX with SFN metabolites inhibits cancer will be helpful for establishing a low-toxicity, low-resistance and high-efficiency anti-cancer therapy.

## Materials and methods

### Reagents

SFN-Cys and SFN-NAC were purchased from Santa Cruz Biotechnology (USA). PTX (Taxol, as a brand name) was obtained from Selleckchem (USA). Anti-Caspase-3, anti-β-actin, anti-α-tubulin, anti-Tau and protein A/G PLUS agarose were purchased from Santa Cruz Biotechnology (USA). Anti-βIII-tubulin and anti-Caspase-7 were purchased from Abcam (USA). Anti-Hsp70, anti-XIAP, anti-ERK1/2 and anti-pERK1/2 (Thr202/Tyr204) were obtained from Cell Signaling Technology (USA). Anti-Stathmin1 was obtained from Sangon Biotech Co.Ltd. (Shanghai, China). Anti-cleaved-PARP and anti-β-tubulin were purchased from Wanleibio (Shenyang, China). Annexin V-FITC/PI apoptosis assay kit was purchased from NeoBioscience (Shenzhen, China). Recombinant human Caspase-3 was purchased from Sino Biological Inc. (Beijing, China).

### Cell culture and cell proliferation assay

A549 cell line was obtained from Cell Resource Center, Peking Union Medical College (CRC/PUMC). All cells were cultured in DMEM/F-12 medium (Lifetechnologies, Shanghai, China) supplemented with 10% fetal bovine serum at 37 ℃ in 5% CO_2_.

A549 cells and A549/Taxol-R cells (4–6 × 10^3^) were plated in 96-well plates. When cells grew up to 80% density, SFN-Cys and SFN-NAC at a series of concentrations were used for 24 h. Cell Proliferation Assay Kit (Promega, Madison, USA) was used to detect the absorbance values of living cells at 490 nm wavelength according to the manufacturer’s instruction.

### Establishment of Taxol-resistant A549 cell line

To establish the Taxol-resistant A549 cells, we treated A549 cells with 20 ng/ml PTX for 24 h, Taxol-sensitive cells generated apoptosis, and the survived A549 cells are collected for further resistant induction. Meanwhile, the concentrations of PTX were used in turn: 20, 40, 60, 80, 100, 120, 200, 300 and 400 ng/ml for 24 h each; the finally survived cells were referred as A549/Taxol-R cells. To confirm that we established a Taxol-resistant cell line successfully, IC50 value was tested, more than 15 of that was regarded to be an index for resistant cell line^10,29^.

### siRNA silencing

To knock down βIII-tubulin and α-tubulin mRNA, βIII-tubulin siRNA (5′-CCCAGCGGCAACTACGTGGG-3′) and α-tubulin siRNA (5′-AAAGATGTCAATGCTGCCATT-3′) were designed^30,31^. Cells were plated in 6-well plates at a density of 1 × 10^6^/well and cultured for 24 h. Then the cells were transfected with the βIII-tubulin and α-tubulin siRNA, respectively (30 pmol/well) by LipofectamineTM RNAiMAX (Invitrogen, USA) when cells reached approximately 80% confluency.

### Cell morphological observation

According to a series of concentrations and times, A549 and A549/Taxol-R cells were treated by either SFN-Cys or SFN-NAC, then cell morphological features were observed with a phase-contrast microscope (Leica, Germany) linked to a digital camera (Olympus, Japan). Harvested cells were processed and the sections were observed and photographed with a transmission electron microscope (TEM, JEM-1400Plus, Japan).

### Apoptosis detection

Cells were treated with SFN metabolites and PTX; the adhesive cells were collected and washed twice with cold phosphate-buffered saline (PBS) and binding buffer. Solution was centrifuged at 1000 g for 7 min and stained with FITC reagent for 30 min as well as PI reagent for 5 min. Cells were analyzed by the flow cytometer (BD Biosciences, Rutherford, NJ).

### Bioinformatics analysis

We searched the GEPIA Database to find the possible correlation between survival rate and expression of microtubule and microtubule related proteins including α-tubulin, βIII-tubulin, Tau, Stathmin1, XIAP, Hsp70 and recorded these results, which are statistically significant (*P* < 0.05)^32^.

### Immunofluorescence staining and confocal microscopy observation

Cells were fixed with 4% paraformaldehyde for 15 min and permeabilized with 0.2% Tween 20 for 10 min. The cells were incubated by primary antibody (anti-βIII-tubulin, 1:200; anti-Tau, 1:100; anti-α-tubulin, 1:400; anti-XIAP, 1:100) for 12 h at 4 ℃ and fluorescence-labeled secondary antibody for 1 h. Finally, the cells were stained with DAPI and observed on confocal laser-scanning microscope (Olympus FV1000, Tokyo, Japan).

### Western blot

Cell lysates were prepared with RIPA lysis buffer (Thermo Fisher Scientific, USA), and protein concentrations were determined by BCA protein assay kit (Invitrogen, USA). Protein was loaded and run through 12% or 15% SDS-PAGE gels and transferred to nitrocellulose membranes. Protein bands were detected by Odyssey infrared imaging system (LI-COR Biosciences, Lincoln, NE, USA).

### Tissue microarray immunohistochemistry

Human lung cancer tissue microarrays with 150 patient samples and different Gleason patterns were established by Shanghai Biochip (Shanghai, China). The protocols came from the reference ^8^.

### Microtubule polymerization assay

The collected cells were washed twice with PBS, then lysed at 37 °C for 30 min with 400 μL lysis buffer (20 mM Tris–HCl, pH 6.8, 1 mM MgCl_2_, 2 mM EGTA, 1% NP-40) with Protease Inhibitor Cocktail (Roche). The cell lysates were centrifuged at 12,000 rpm for 15 min at 25 °C. The supernatant containing soluble α-tubulin (depolymerization/free) was collected, while the pellet containing assembled α-tubulin (polymerization/microtubule) was suspended in 40 μL of pellet lysis buffer (20 mM Tris–HCl, pH 6.8, 1 mM MgCl_2_, 2 mM EGTA, 2% SDS) after washed with PBS. Then, the precipitate was heated at 100 °C for 30 min until the pellet was solved. The α-tubulin proteins in two fractions (soluble and insoluble) were separated by Western blot.

### Co-immunoprecipitation

Cells were plated at a density of 5 × 10^6^ cells/dish and cultured for 24 h. Then the cells were treated with SFN-Cys (30 μM) and SFN-NAC (30 μM) for 24 h, and washed with ice-cold PBS, then lysed on ice via Nondenaturing Lysis Buffer (APPLYGEN, China) with protease inhibitors cocktail. The cell lysates were incubated with the corresponding antibody overnight at 4 °C. The complexes were pulled down with protein A/G agarose for 3 h and the proteins were isolated by centrifuging and boiling for 5 min. Western blot was used to recognize the conjugated proteins.

### Caspase-3 cleavage assay

Cells were treated with 30 μM either SFN-NAC or SFN-Cys for 24 h and the harvested cells were lysed in Pierce RIPA Buffer (Thermo scientific, USA) and 12 μg cell extract was incubated with 4 μL recombinant Caspase-3 (Sino Biological Inc.), 5 μL 100 mM DTT in 50 μL reaction buffer containing 25 mM Hepes, pH 7.5, 0.1% Chaps, at 37 °C for 6 h. After incubation, Western blot analysis was used to detect the degradation of α-tubulin.

### Statistical analysis

Data were expressed as mean ± standard deviation. Paired data were evaluated by Mann Whitney test, and two groups were compared by Student t test. Statistical significance was determined at the 0.05 or 0.01 level. The statistical analyses were done by SPSS version 19.0.

## Results

### Establishment of Taxol-resistant cell line with increasing expression of microtubule associated proteins and Hsp70

**Fig. 1:**
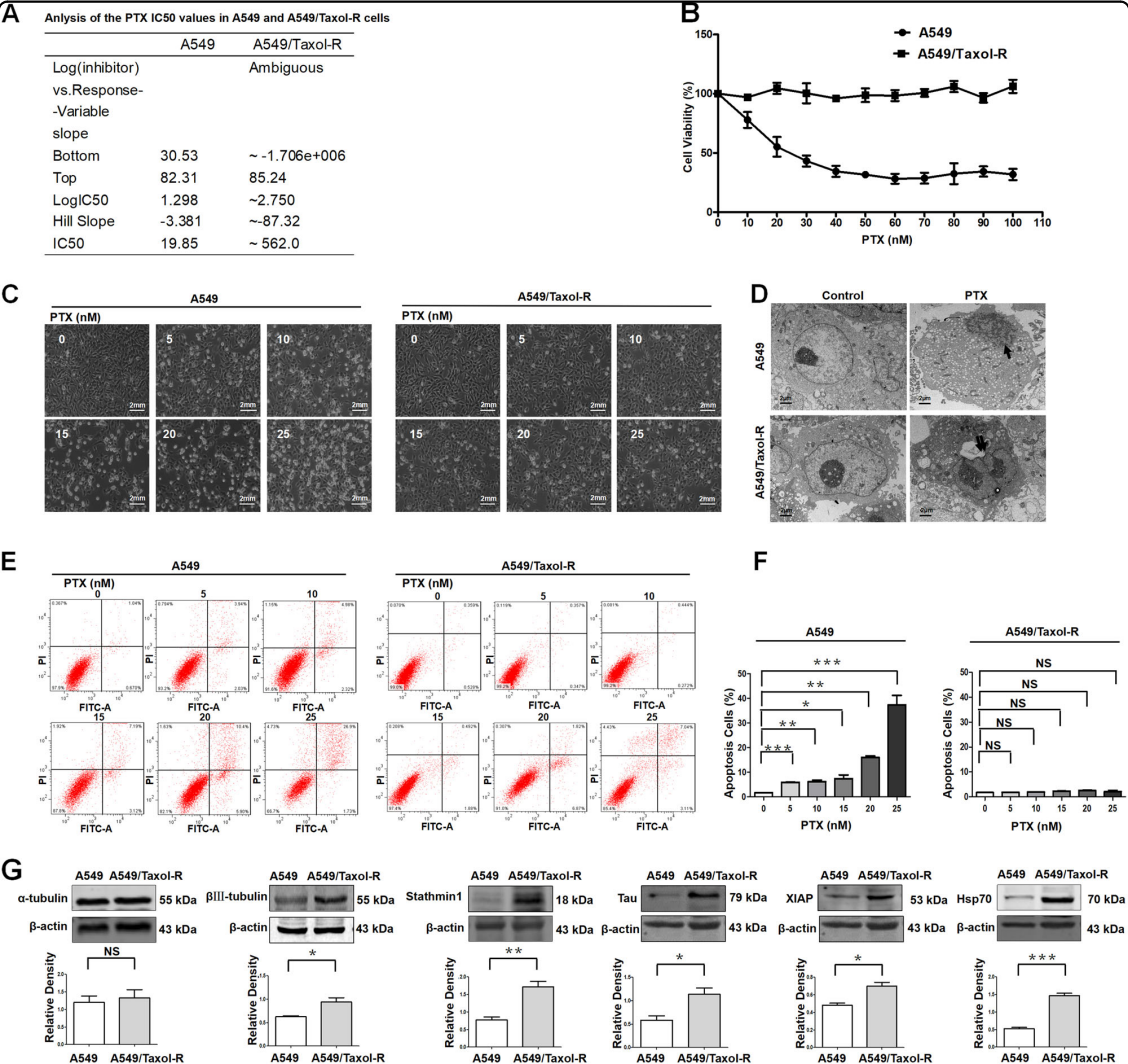
Establishment of PTX-resistant cell line A549/Taxol-R. **a** Both A549 cells and A549/Taxol-R cells treated with PTXs were analyzed for IC50 by Graphpad prism 5 software. **b** Both A549 and A549/Taxol-R cells were treated with gradient-concentrations of PTX for 24 h. Then, cell viability was determined by Cell Proliferation Assay Kit. Cell viability (percentage) was ratio of OD at 490 nm value of each group cells vs. OD value of control group cells. **c** Both A549 cells and A549/Taxol-R cells were treated with 0, 5, 10, 15, 20, 25 nM PTX and recorded by Leica DMIRB microscope at × 40 magnification for 24 h. **d** After treated with 20 nM PTX, both A549 and A549/Taxol-R cells were harvested and finally were viewed under TEM. Black arrow indicates nucleic fragmentation, and double black arrows indicate mitochondria. **e** Both A549 and A549/Taxol-R cells were treated with 0, 5, 10, 15, 20, 25 nM for 24 h, then the cells were harvested and the percentage of cell apoptosis was analyzed by flow cytometry via Annexin V-FITC/PI Apoptosis Detection Kit. **f** The histogram showed the quantification of apoptosis cells of A549 and A549/Taxol-R cells. These results were from three independent experiments. **g** The expressions of βIII-tubulin, XIAP, Tau, Stathmin1, Hsp70 and α-tubulin were detected by Western blot in A549 cells and A549/Taxol-R cells. Data were shown as means ± SD from three separate experiments. *P < 0.05; **P < 0.01; ***P < 0.001, *NS* no significance vs. control group, *n* ≥ 3

First, we established a cell line, named A549/Taxol-R. The resistance index is 28.31, namely ratio of IC50 of A549/Taxol-R cells vs. IC50 of A549 cells. (Fig. 1a). Cell proliferation assay showed that A549/Taxol-R cells were resistant to PTX after gradient induction compared with A549 parental cells (Fig. 1b). Cell morphological features changed in response to PTX. Results showed that parental A549 cells got shrunk from normal spindle-shaped to round and transparent shapes with short processes compared with A549/Taxol-R cells (Fig. 1c). In response to 20 nM PTX for 24 h, the A549 cells exhibited nuclear fragmentation (black arrow), but the A549/Taxol-R cells exhibited double nuclei (double arrow) under TEM (Fig. 1d). Meanwhile, flow cytometry assay showed that the increasing concentrations of PTX induced apoptosis in the A549 cells, but not in A549/Taxol-R cells (Fig. 1e). Western blot results showed that the expressions of βIII-tubulin, XIAP, Stathmin1, Tau, Hsp70, except α-tubulin in A549/Taxol-R cells were significantly higher than those in A549 cells (Fig. 1g).

### High expression of βIII-tubulin in human NSCLC tissues showed positive correlations with cancer malignant grading

**Fig. 2:**
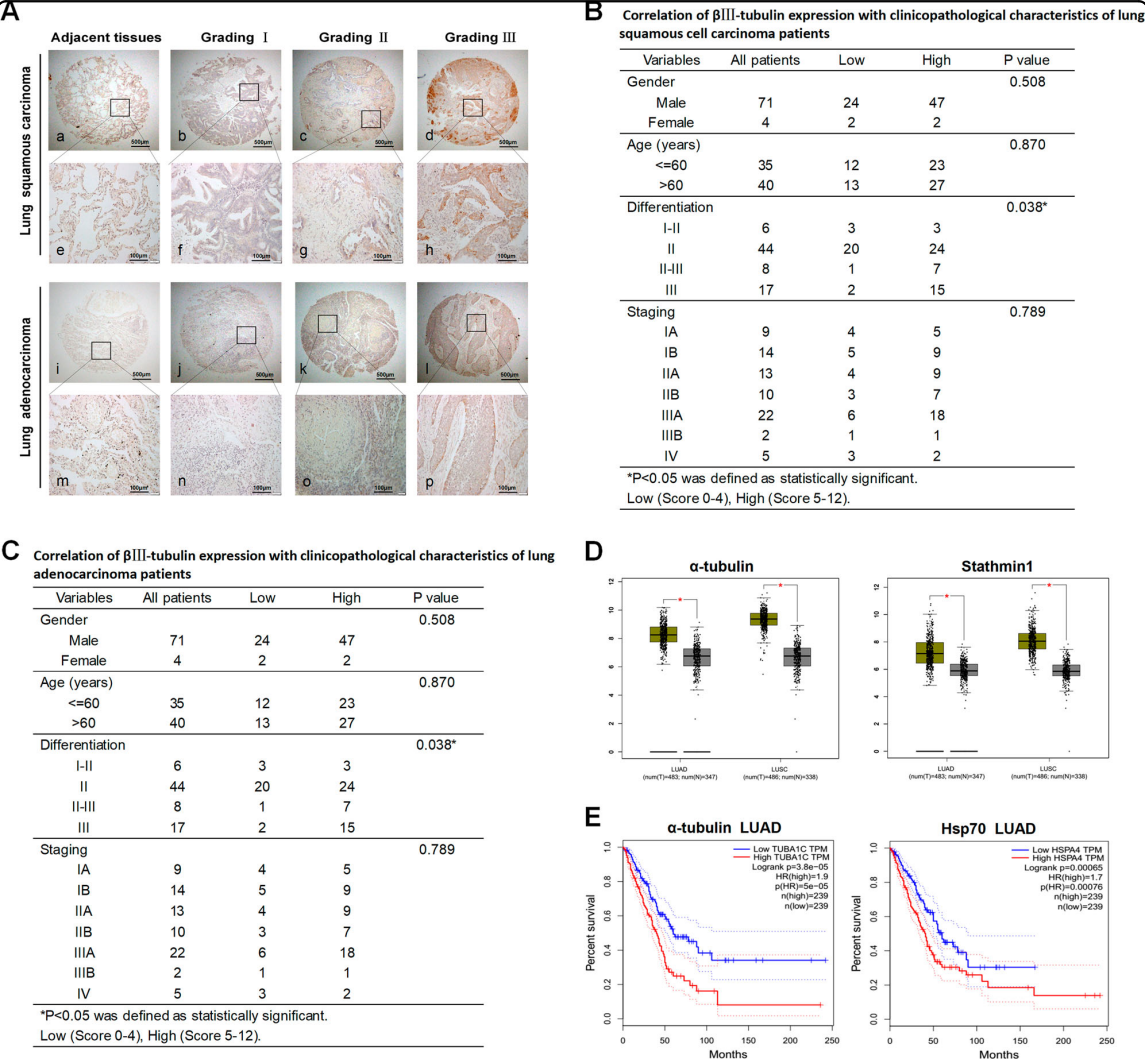
βIII-tubulin expression showed positive correlation with pathological grading in NSCLC tissues and survival analysis by GEAPIA Database. **a** βIII-tubulin was expressed in human NSCLC tissues associated with various histopathological grading by IHC staining. The expression of βIII-tubulin in the adjacent tissues was observed as the control (**a**, **e**, **i**, **m**). IHC, magnification × 40, in **a**–**l**; magnification × 200, in **e**–**p**. **b** The correlation of βIII-tubulin expression with clinicopathological characteristics of lung squamous cell carcinoma patients. H scores (low: 0–4; high: 5–12). **c** The correlation of βIII-tubulin expression with clinicopathological characteristics of lung adenocarcinoma patients. H scores (low: 0–4; high: 5–12). **d** The expression of drug resistance-related protein α-tubulin and Stathmin1 in tumor patients and normal adults. LUAD (lung adenocarcinoma), LUSC (lung squamous carcinoma), T (tumor patients), N (normal adults). **e** The survival analysis of drug resistance-related protein α-tubulin and Hsp70 via GEAPIA Database^32^. Data were shown as *P < 0.05; **P < 0.01; ***P < 0.001, *NS* no significance vs. control group, *n* ≥ 3

We detected highly expressed α-tubulin and Hsp70 via immunohistochemistry staining^7^ on tissue arrays of human lung adenocarcinoma or lung squamous carcinoma samples. Similarly, we detected the overexpression of βIII-tubulin. The results showed that the stained intensity of tumor tissues with βIII-tubulin were raised as the pathological grading increased, while those in the adjacent tissues were not significantly stained. Specifically, the H-scores of tumor tissues by IHC staining with βIII-tubulin were higher than those of adjacent tissues (Fig. 2a). Results showed that the ratio of samples with high expression of βIII-tubulin was found in 3 out of 6 lung squamous cell carcinoma samples (50%) with grading I-II, and in 24 of 44 samples (54.5%) with grading II, in 7 of 8 samples (87.5%), with grading II-III, in 15 of 17 samples (88.2%) with grading III (Fig. 2b, *P* = 0.038). Results showed that the ratio of samples with high expression of βIII-tubulin was found in 6 out of 14 lung adenocarcinoma cell carcinoma samples (42.9%) with grading I-II, and in 23 of 44 samples (52.3%) with grading II, in 11 of 13 samples (84.6%) with grading II-III, while in 4 of 4 samples (100%) with grading III (Fig. 2c, P = 0.037). These findings suggested that βIII-tubulin overexpression may be required for the maintenance of tumor malignant phenotypes. We searched the GEPIA Database to find bioinformatics information that α-tubulin and Stathmin1 expressed significantly higher in either squamous carcinoma or adenocarcinoma of lung than those in normal tissues. (Fig. 2d). Also, survival analysis showed that patients with low expression α-tubulin and Hsp70 had higher survival rate, indicating that α-tubulin and Hsp70 might be the tumor proliferation and resistance-promoting factors (Fig. 2e).

### SFN metabolites induced apoptosis via downregulating microtubule associated proteins and upregulating Hsp70

**Fig. 3:**
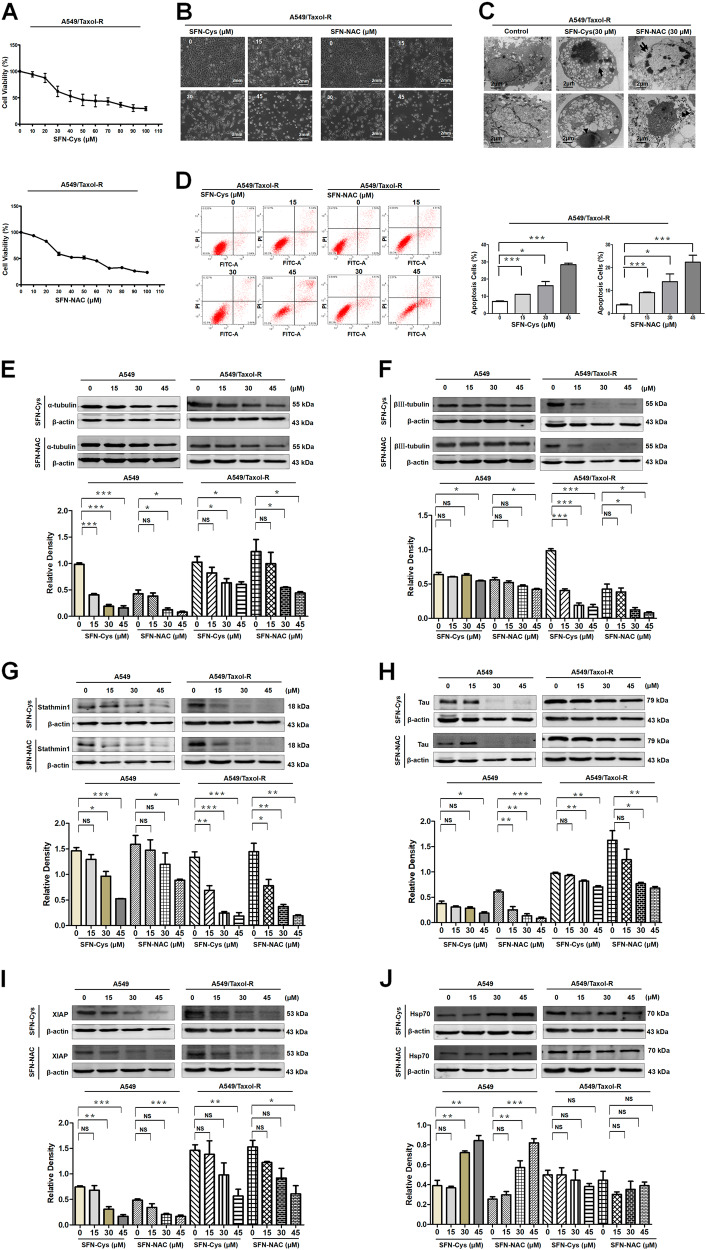
SFN metabolites induced apoptosis via downregulating microtubule associated proteins and upregulating Hsp70 in A549/Taxol-R cells. **a** A549/Taxol-R cells were treated with either SFN-Cys or SFN-NAC (0, 10, 20, 30, 40, 50, 60, 70, 80, 90, 100 μM) at the indicated concentrations for 24 h. Then, cell viability was determined by Cell Proliferation Assay Kit. **b** A549/Taxol-R cells were treated with either SFN-Cys or SFN-NAC (0, 15, 30, 45 μM) and recorded by Leica DMIRB microscope at × 40 magnification for 24 h. **c** After treated with either SFN-Cys or SFN-NAC (30 μM) for 24 h, A549/Taxol-R cells were harvested and were viewed with a transmission electron microscope. Black arrow indicates sporadic vacuoles, double lack arrows indicate nucleic condensation like a flower ring, arrow head indicates karyopyknosis, double arrow heads indicate apoptotic body. **d** A549/Taxol-R cells were treated with either SFN-Cys or SFN-NAC (0, 15, 20, 30 μM) for 24 h, the percentage of cell apoptosis was analyzed by flow cytometry via Annexin V-FITC/PI Apoptosis Detection Kit. **e**–**j** The expression of α-tubulin, βIII-tubulin, Stathmin1, Tau, XIAP, Hsp70 was detected by Western blot with the treatment of either 0, 15, 30, 45 μM SFN-Cys or SFN-NAC in bothA549 and A549/Taxol-R cells. Data were shown as means ± SD from three separate experiments. *P < 0.05; **P < 0.01; ***P < 0.001, vs. control group, *NS* no significance, *n* ≥ 3

Cell proliferation assay showed that SFN-Cys or SFN-NAC reduced cell viability in a dose-dependent manner in A549/Taxol-R cells (Fig. 3a). The optimal concentration of SFN metabolites sensitive to A549/Taxol-R cells is 30 μM. Treatment with SFN-Cys or SFN-NAC for 24 h, A549/Taxol-R cells became round, shrunk with short processes, even dead (Fig. 3b). Under TEM, the cells exhibited nuclear fragments (black arrow) and flower-like rings (double arrows), nucleic condensation (arrow head), sporadic vacuoles and apoptotic bodies (double arrow heads) (Fig. 3c). More, flow cytometry assay showed that SFN metabolites induced apoptosis in a concentration-dependent manner in A549/Taxol-R (Fig. 3d). Western blot results showed that SFN metabolites downregulated microtubule-related proteins in a dose-dependent manner. Those proteins including βIII-tubulin, XIAP, Tau, Stathmin1 and α-tubulin were downregulated, while Hsp70 was upregulated gradually in both A549/Taxol-R and A549 cells after treated with either SFN-Cys (0, 15, 30, 45 μM) or SFN-NAC (0, 15, 30, 45 μM) (Fig. 3f–k).

### SFN metabolites upregulated 26S proteasome via sustained ERK1/2 phosphorylation to degrade resistance-related proteins

**Fig. 4:**
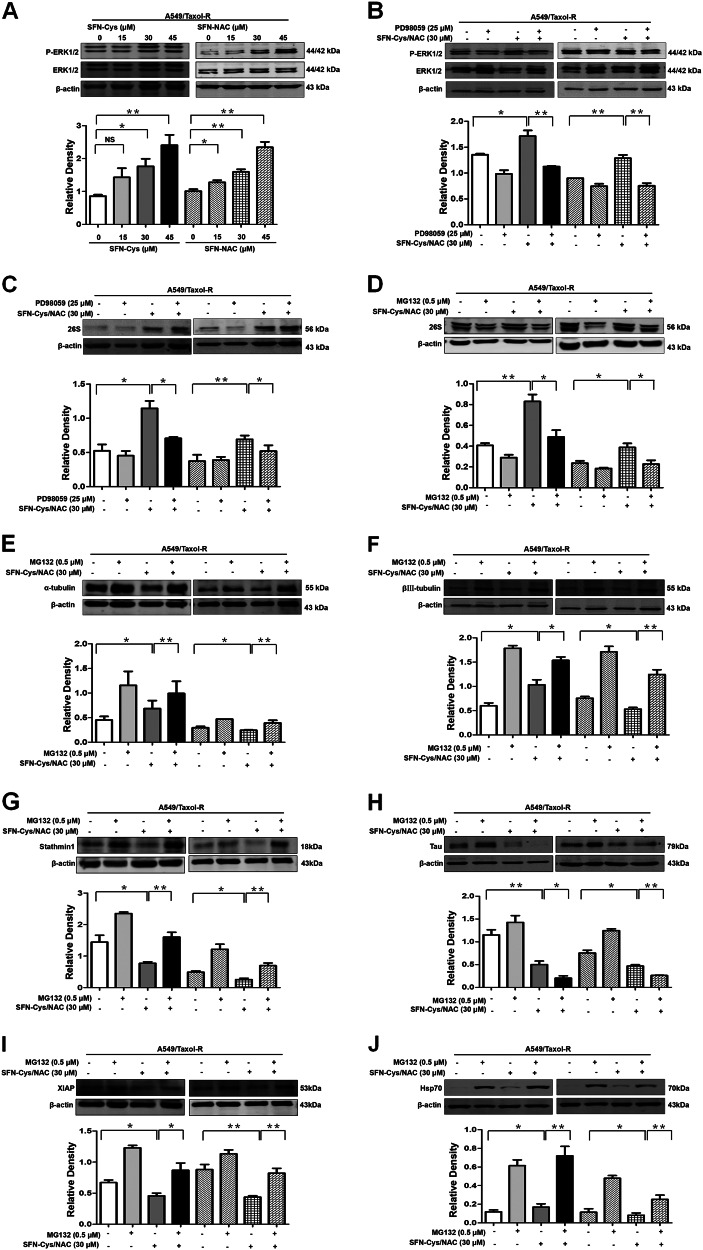
SFN metabolites upregulated 26S proteasome via sustained ERK1/2 phosphorylation to degrade resistance-related proteins. **a** The expression of ERK1/2, phosphorylated ERK1/2 (pERK1/2) was detected by Western blot with the treatment of 0, 15, 30, 45 μM either SFN-Cys or SFN-NAC for 24 h in A549/Taxol-R cells. **b** The expression of ERK1/2 and pERK1/2 was detected by Western blot with the treatment of either 30 μM SFN-Cys or 30 μM SFN-NAC with/without 25 μM PD98059 for 24 h in A549/Taxol-R cells. **c** The expression of 26 S was detected by Western blot with the treatment of either 30 μM SFN-Cys or 30 μM SFN-NAC with/without 25 μM PD98059 for 24 h in A549/Taxol-R cells. **d** The expression of 26 S was detected by Western blot with the treatment of either 30 μM SFN-Cys or 30 μM SFN-NAC with/without 0.5 μM MG132 (0.5 μM) for 24 h in A549/Taxol-R cells. **e**–**j** The expression of α-tubulin, βIII-tubulin, Stathmin1, Tau, XIAP, Hsp70 was detected by Western blot with the treatment of either 30 μM SFN-Cys or 30 μM SFN-NAC with/without 0.5 μM MG132 for 24 h in A549/Taxol-R cells. Data were shown as means ± SD from three separate experiments. *P < 0.05; **P < 0.01; ***P < 0.001, *NS* no significance vs. control group, *n* ≥ 3

Here we discovered that either SFN-Cys or SFN-NAC persistently induced the phosphorylation of ERK1/2 following a concentration-dependent manner (Fig. 4a). These effects can be blocked by the pERK1/2 inhibitor PD98059 (25 μM) (Fig. 4b). Further, SFN-Cys or SFN-NAC upregulated 26 S proteasome resulting from phosphorylated ERK1/2 (Fig. 4c, d). Proteasome blocker, MG132 (0.5 μM) was used to demonstrate that downregulation of βIII-tubulin, α-tubulin, XIAP, Tau and Stathmin1, resulted from 26S proteasome activation. (Fig. 4e–h).

### SFN metabolites lowered the interaction among microtubule associated proteins leading to microtubule disruption and reduced resistance to PTX

**Fig. 5:**
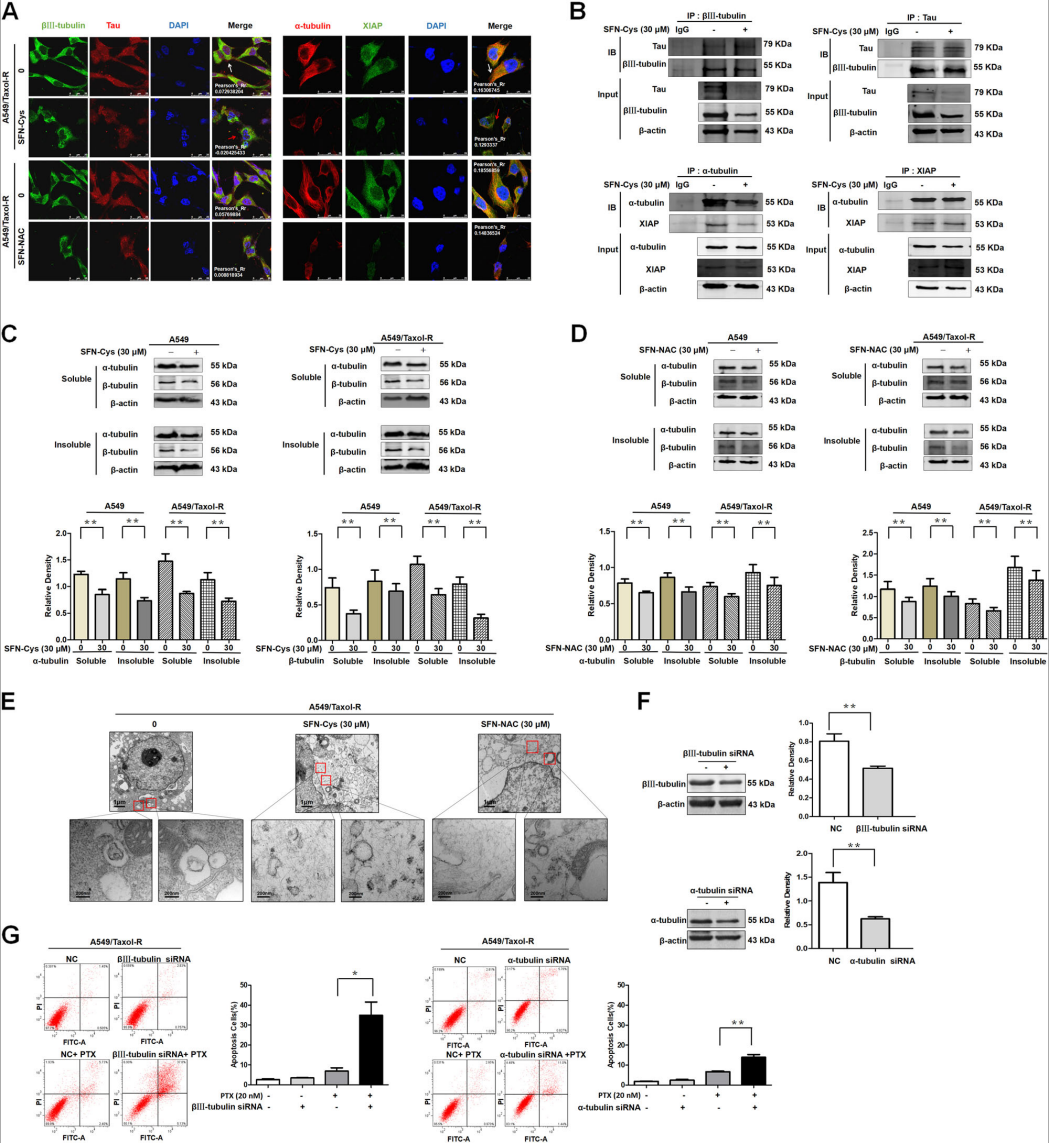
SFN metabolites lowered the interactions among microtubule associated proteins leading to microtubule disruption and reduced resistance to PTX. **a** Immunofluorescence staining of Tau, α-tubulin and βIII-tubulin, XIAP showed the raising co-localization in cells and the changes of microtubule morphology treated with either 30 μM SFN-Cys or 30 μM SFN-NAC. Blue: DAPI-stained DNA; White arrows: normal microtubules; red arrows: the abnormal microtubules. Scale bars, 25 μm. The images in last row exhibited the zoom-in merged results. **b** Cells were treated with 30 μM SFN-Cys for 24 h. The binding of Tau to βIII-tubulin and binding of XIAP to α-tubulin was detected in A549/Taxol-R cells by forward and reverse co-immunoprecipitation (Co-IP). β-actin was used to be the loading controls for input proteins. **c** The dynamics of microtubules was measured by microtubule polymerization assay in vivo, and β-actin acted as the loading control. The expression of α-tubulin and β-tubulin was detected by Western blot with the treatment of 30 μM SFN-Cys for 24 h in A549/Taxol-R cells in soluble and insoluble cell lysate. The histogram showed the quantification of soluble and insoluble α-tubulin and β-tubulin. These results were from three independent experiments. **d** The expression of α-tubulin and β-tubulin was detected by Western blot with the treatment of 30 μM SFN-NAC for 24 h in A549/Taxol-R cells in soluble and insoluble cell lysate. The histogram showed the quantification of soluble and insoluble α-tubulin and β-tubulin. These results were from three independent experiments. **e** After treated with either 30 μM SFN-Cys or 30 μM SFN-NAC 24 h in A549/Taxol-R cells, then the cells were harvested and fixed, the cells pellets were cut into thin slices and microtubule structures were observed with a transmission electron microscope. **f** The expression of βIII-tubulin and α-tubulin was detected by Western blot after knockdown of βIII-tubulin and α-tubulin via siRNA in A549/Taxol-R cells. **g** Knockdown of βIII-tubulin and α-tubulin via RNA interference in A549/Taxol-R cells with/without 20 nM for 24 h, then the cells were harvested and the percentage of cell apoptosis was analyzed by flow cytometry via Annexin V-FITC/PI Apoptosis Detection Kit. Data were shown as means ± SD from three separate experiments. *P < 0.05; **P < 0.01; ***P < 0.001, *NS* no significance vs. control group, *n* ≥ 3

After the cells were treated with 30 μM SFN-Cys or 30 μM SFN-NAC, immunofluorescence and confocal microscopy analysis showed the colocalization of Tau/βIII-tubulin and XIAP/α-tubulin (Fig. 5a). Meanwhile, co-immunoprecipitation showed that SFN-NAC reduced the interaction of Tau/βIII-tubulin and XIAP/α-tubulin (Fig. 5b). Further, both soluble and insoluble α-tubulin were decreased and the similar results were obtained in βIII-tubulin detection (Fig. 5c, d). These indicated that SFN metabolites induced microtubule depolymerization. Under confocal microscope, we observed nest-like microtubule structures in A549/Taxol-R cells treated with 30 μM SFN-Cys or 30 μM SFN-NAC for 24 h, the microtubule features exhibited morphological disorders and crinkles like broken filaments (Fig. 5e). Interestingly, using either α-tubulin siRNA or βIII-tubulin siRNA we knocked down α-tubulin or βIII-tubulin in A549/Taxol-R cells (Fig. 5f), flow cytometry showed that A549/Taxol-R cells got more sensitive to 20 nM PTX (Fig. 5g).

### Combination of PTX and SFN metabolites showed a synergistic inhibition

**Fig. 6:**
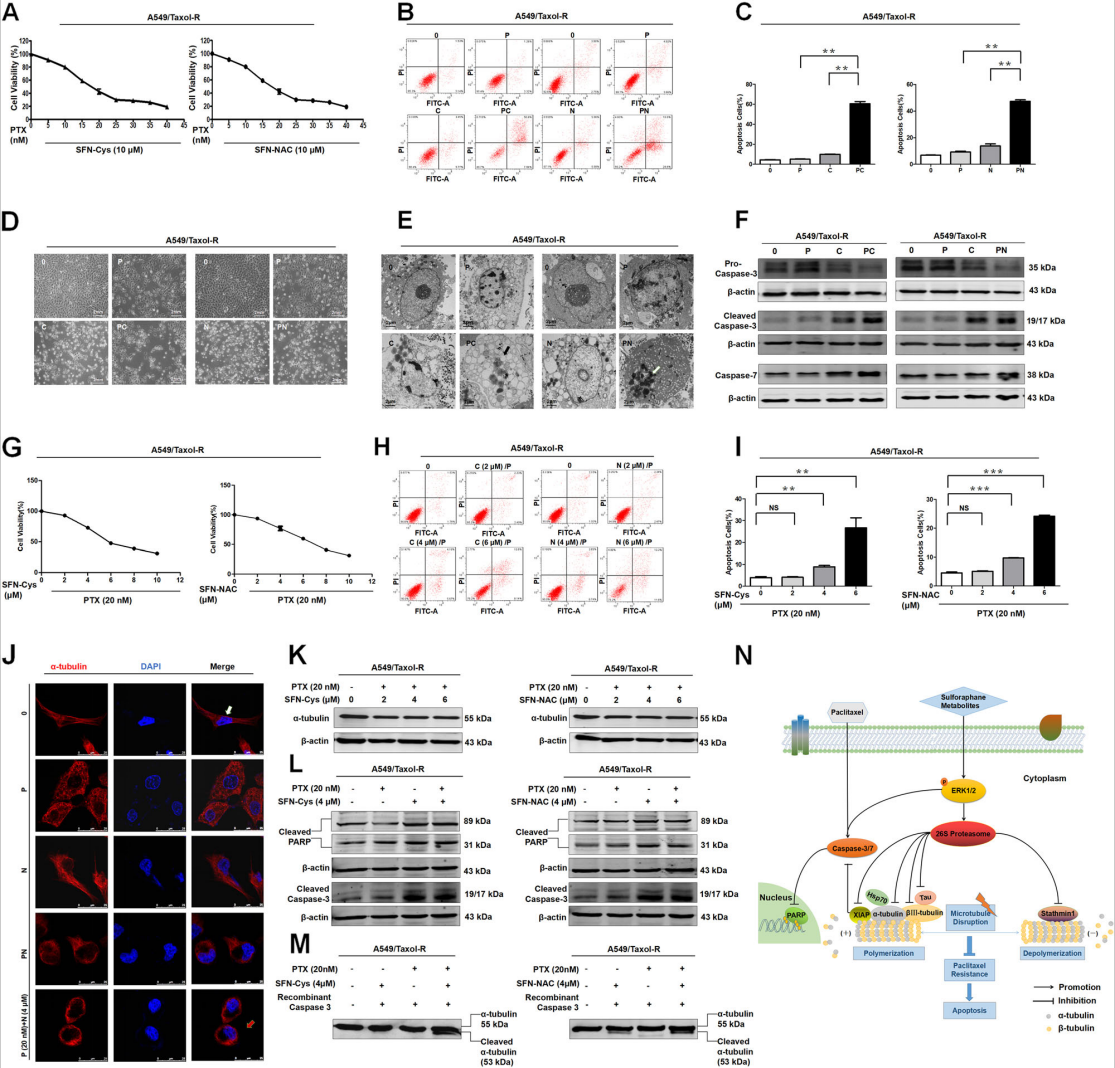
Combination of PTX with SFN metabolites showed a synergistic inhibition in A549/Taxol-R cells. **a** A549/Taxol-R cells were treated with (0, 5, 10, 15, 20, 25, 30, 35, 40 nM) combined with either 10 μM SFN-Cys or 10 μM SFN-NAC, respectively at the indicated concentrations for 24 h. Then, cell viability was determined by Cell Proliferation Assay Kit. **b** A549/Taxol-R cells were treated with PTX (20 nM), SFN-Cys (20 μM) or SFN-NAC (20 μM), SFN-Cys (10 μM) or SFN-NAC (10 μM) combined with PTX (10 nM), respectively for 24 h, then the cells were harvested and the percentage of cell apoptosis was analyzed by flow cytometry via Annexin V-FITC/PI Apoptosis Detection Kit (P: PTX 20 nM; C: SFN-Cys 20 μM; N: SFN-NAC 20 μM; PC: PTX 10 nM + SFN-Cys 10 μM; PN: PTX 10 nM + SFN-NAC 10 μM). **c** The histogram demonstrated the number of apoptotic cells in each group was detected by flow cytometry. **d** A549/Taxol-R cells was treated with both PTX (20 nM), SFN-Cys (20 μM) or SFN-NAC (20 μM), SFN-Cys (10 μM) or SFN-NAC (10 μM) combined with PTX (10 nM), respectively for 24 h, then recorded by Leica DMIRB microscope at ×40 magnification. **e** A549/Taxol-R cells were treated with PTX (20 nM), SFN-Cys (20 μM) or SFN-NAC (20 μM), SFN-Cys (10 μM) or SFN-NAC (10 μM) combined with PTX (10 nM) respectively for 24 h, then we harvested cells and viewed subcellular structures with a transmission electron microscope. Black arrows indicated sporadic vacuoles, white arrows indicated nucleic condensation. **f** The expression of Caspase-7, pro-Caspase-3 and cleaved-Caspase-3 was detected by Western blot in the groups (P: PTX 20 nM; C: SFN-Cys 20 μM; N: SFN-NAC 20 μM; PC: PTX 10 nM + SFN-Cys 10 μM; PN: PTX 10 nM + SFN-NAC 10 μM). **g** Cell viability was determined by Cell Proliferation Assay Kit. **h** Cells were harvested and the percentages of cell apoptosis were analyzed by flow cytometry via Annexin V-FITC/PI Apoptosis Detection Kit. **i** The histogram showed the number of apoptotic cells in each group. **j** Immunofluorescence staining of α-tubulin showed the changes of microtubule morphology treated with PTX and SFN-NAC in different groups. We also treated A549/Taxol-R cells with PTX and SFN-Cys in different groups, the results are the same as the combination of PTX and SFN-NAC (data not shown). Red: α-tubulin, Blue: DAPI-stained DNA; White arrows: normal microtubules; red arrows: the abnormal microtubules. Scale bars, 25 μm. **k** The expression of α-tubulin was detected by Western blot in each group of A549/Taxol-R cells. **l**: PARP has a molecular weight of 116 kDa and was cleaved into 89 and 31 kDa fragments by activated Caspase-3 in each group of A549/Taxol-R cells, the expression of cleaved-Caspase-3 was detected by Western blot in the above groups. **m** Recombinant Caspase-3 cleaved α-tubulin only in the combined treatment other than single treatment and cleaved-α-tubulin was an approximately 53 kDa fragment. **n** A schematic of the involved signal pathways that SFN metabolites and PTX disturbed microtubule dynamics and activated the intrinsic apoptosis pathway leading to apoptosis in A549/Taxol-R cells. Data were shown as means ± SD from three separate experiments. *P < 0.05; **P < 0.01; ***P < 0.001, *NS* no significance vs. control group, *n* ≥ 3

PTX combined with SFN metabolites (10 μM) was used to reduce cell viability efficiently in A549/Taxol-R cells (Fig. 6a). Flow cytometry assay showed that the combination of PTX (10 nM) with SFN metabolites (10 μM) caused synergistic effects compared with either PTX (20 nM) or SFN metabolites (20 μM) only (Fig. 6b). PTX combined with SFN metabolites synergistically induced apoptosis in A549/Taxol-R cells. After treated with SFN-Cys or SFN-NAC, A549/Taxol-R cells became round, the processes got shorter. The number of cell apoptosis increased significantly in the cells treated with the either 20 nM PTX or 20 μM SFN metabolites only (Fig. 6d). Under TEM, we observed that the cells exhibited more cytoplasmic vacuoles, nuclear agglutinations and fragmentations in the cells treated with combined drugs (Fig. 6e). SFN metabolites activated Caspase-3 via the ERK1/2 pathway, while PTX also activated Caspase-3^33^. Western blot results showed that cleaved-Caspase-3 and Caspase-7 increased significantly in the cells treated with PTX (10 nM) with SFN metabolites (10 μM) vs. either PTX (20 nM) or SFN metabolites (10 μM) only (Fig. 6f). To find a minimum dose of SFN metabolites which took effect, we used 20 nM PTX combined with a series of concentrations SFN metabolites to test the reduced cell viability in A549/Taxol-R cells via cell proliferation assay kit. Results showed that 20 nM PTX combined a minimum 4 μM SFN metabolites caused a significant inhibition of cell proliferation (Fig. 6g). Flow cytometry assay also showed that the combination caused synergistic effects (Fig. 6h, i). Immunofluorescence assay showed that α-tubulin fluorescence was decreased; microtubule was disrupted more significantly in the combination of PTX 20 nM with 4 μM SFN metabolites (Fig. 6j). Further, Western blot showed that the combination induced significant downregulation of α-tubulin (Fig. 6k) and increased cleaved PARP by Caspase-3 cleavage than that by either SFN metabolites (4 μM) or PTX (20 nM) only (Fig. 6l). Caspase-3 cleavage assay showed that α-tubulin was cleaved and produced an approximately 2 kDa cleaved α-tubulin in response to the combined drugs only (Fig. 6m).

SFN metabolites downregulated microtubule proteins and microtubule related proteins via activating 26S proteasome resulting in microtubule disruption. These proteins were also the main proteins that increased drug resistance to PTX. PTX was a microtubule stabilizer and interfering agent, and SFN metabolites depolymerized microtubules, and their combination resulted in a synergistic imbalance of microtubule dynamics to promote cell apoptosis. Both of them activated Caspase-3 to cleave PARP and α-tubulin leading to apoptosis through the intrinsic apoptotic pathways (Fig. 6n).

## Discussion

The extensive application of PTX brings gospel to cancer sufferers. However, the increased resistance to PTX is the main culprit of therapeutic failure. Even worse, PTX might induce neoplasm metastasis and produce various side-effects such as cell toxicity, hematopoietic function inhibition and immunity reduction in patients^33,34^. Therefore, it is crucial to lower this drug resistance via establishing an innovated therapy. Numerous studies showed that SFN and its metabolites inhibit tumor growth, angiogenesis, invasion and metastasis, and induce apoptosis^8,22,35,36^. More recently, we found that SFN metabolites induce apoptosis via triggering microtubule disruption and inhibiting Hsp70-mediated autophagy in cancer cells^7^. Here we further discovered that SFN metabolites induced apoptosis in PTX-resistant cells via phosphorylated ERK1/2-mediated upregulation of 26 S proteasome and downregulation of βIII-tubulin, XIAP, Tau, Stathmin1 and α-tubulin. Upregulation of Hsp70 was considered as a feedback of tumor cells after SFN metabolites treatments. It was reported that inhibition of autophagy increased paclitaxel sensitivity^37,38^. We believed that inhibition of Hsp70-mediated autophagy via SFN metabolites might sensitize PTX to cancer cells. These findings indicated that SFN metabolites might increase PTX sensitivity to PTX-resistant cells. Interestingly, we further demonstrated that combination of PTX and SFN metabolites lowered resistance to PTX and anti-cancer doses of PTX and SFN metabolites. Especially, SFN metabolites work greatly at a lower dose (4 μM) by drug combination, which solved the problem that SFN metabolite only could not be used for clinical trial because of higher anti-cancer concentration.

Accumulating studies showed that microtubule associated proteins regulate PTX sensitivity in a wide range of cancer types^12,13,15–17^. Microtubules consist of α- and β-tubulin heterodimers aligned in a head-to-tail pattern^39^. Alterations in the expression of β-tubulin isotypes and apoptotic regulatory proteins such as XIAP and MAPs (microtubule-associated proteins) regulated PTX activity in different types of cancers^12,15,20,30^. For example, drug-resistant cancer cells and human tumor tissues were shown to harbor tubulin gene mutations, alterations in total tubulin content, altered microtubule polymer levels, altered expression of tubulin isotypes, and altered microtubule-associated protein expression^13,14,21,28,30^. High expression of βIII-tubulin has been found to be correlated either with low response rates in patients treated with regimens containing taxanes or vinorelbine or with reduced survival in patients with NSCLC, breast, ovarian, and gastric cancers^40^. The present results showed that expression of βIII-tubulin was significantly related to pathological grading of NSCLC patients. We reported that high expression of α-tubulin and Hsp70 was correlated to NSCLC malignant grading^7^. Hsp70 is a microtubule mediator and resistance promoter^21^. Here, bioinformatics analysis showed that the expression of α-tubulin and Stathmin1 was significantly higher in either squamous carcinoma or adenocarcinoma tissues of lung than that in normal tissues. More, the patients with low expression α-tubulin and Hsp70 had higher survival rate. These data indicated that we might improve the survival rate and prognosis of patients through downregulation of Stathmin1, α-tubulin and Hsp70 leading to microtubule dysfunction. Tau binds to β-tubulin in the same site as PTX, and consequently competes with the PTX^41,42^. An in vitro study showed that preincubation of tubulin with Tau decreased PTX binding and reduced PTX-induced microtubule polymerization in breast cancer cells^18^. In addition, high expression of Tau showed a significant association with poor response to PTX chemotherapy in patients with metastatic breast cancer^43^. Besides, studies showed that overexpression of Stathmin1 decreased sensitivity to PTX. Knockdown of Stathmin1 improved sensitivity to the tubulin-targeting drugs PTX and vinblastine in esophageal squamous cell carcinoma^44^. In the A549/Taxol-R cells we established, the expressions of βIII-tubulin, XIAP, Tau, Stathmin1 and Hsp70 other than α-tubulin were increased simultaneously compared with A549 cells. Here, knockdown of βIII-tubulin and α-tubulin via siRNA increased cell sensitivity to PTX in PTX-resistant cells. Accordingly, SFN-Cys and SFN-NAC lowered resistance to PTX via downregulating βIII-tubulin, α-tubulin, XIAP, Tau, Stathmin1 leading to microtubule disruption.

Studies showed that sustained ERK1/2 phosphorylation by SFN induced apoptosis^45^, but transient ERK1/2 activation contributed to cancer proliferation in vitro^46^. We found that the phosphorylated ERK1/2 inhibitor PD98059 and proteasome inhibitor MG132 successfully blocked the upregulation of pERK1/2 and 26 S proteasome activation via SFN metabolites. Report showed that SFN activated ERK1/2 persistently to modify specific 26 S proteasomal subunits leading to degradation of target protein^22^. Therefore, phosphorylated ERK1/2 regulated the downstream signaling molecules leading to microtubule disruption.

Previous studies showed that SFN selectively induced degradation of both β-tubulin and α-tubulin in a variety of human cancer cell lines in a dose- and time-dependent manner^24^. The degradation was a proteasome-dependent, rapid and irreversible process initiated by tubulin aggregation^24^. Some studies showed that XIAP, Tau, Hsp70 partly were degraded by 26S proteasome^25–28^. Here SFN-Cys and SFN-NAC induced the degradation of XIAP, Tau, Hsp70, Stathmin1 via upregulating 26S proteasome, and the effects of degradation were reversed by 26S proteasome inhibitor MG132.

Mounting evidence showed that dysregulation of microtubule dynamics contributed to the development of various cancers and occurrence of drug resistance to PTX^18,47^. Here we determined that SFN metabolites reduced insoluble α-tubulin and free α-tubulin resulting in microtubule disassembly and generation of ‘nest-like’ structures of microtubule distribution, which were consistent with those results in the immunofluorescence assay. Consequently, SFN metabolites broke the dynamic balance of microtubules leading to microtubule disruption and reduced drug resistance. Meanwhile, we found that SFN metabolites reduced the interaction between Tau and βIII-tubulin. Apart from downregulating βIII-tubulin, SFN metabolites might reduce the resistance to PTX via diminishing the competition of Tau with PTX in binding to βIII-tubulin. Interestingly, XIAP bound to α-tubulin and SFN metabolites lowered the interaction between XIAP and α-tubulin. Both XIAP and α-tubulin were degraded by 26S proteasome^48^. This remodeling effect was mediated by the ubiquitination and degradation of XIAP and α-tubulin. Also, decrease of XIAP levels reduced the inhibition of Caspase-3 activation and increased microtubule disruption^48^.

Besides, PTX has poor water solubility and the solvents for injection might produce severe side-effects such as hypersensitivity, neutropenia myelosuppression, neutropenia, and neurotoxicity^49^, whereas SFN is a fat-soluble chemical, the combination of two drugs might promote formulations of non-injection administration of PTX via a SFN-enabled self-microemulsifying delivery system^50^. Except the ubiquitin-proteasome system activation, Caspase-3 cleavage might be an extra degrading way to cleave microtubule or its associated proteins. Further, Caspases might cleave not only microtubule proteins, such as α-tubulin but also Tau, Drebrin and Spinophilin, etc^8^. Here, we detected that Caspase-3 cleaved α-tubulin selectively other than βIII-tubulin. Combined PTX and SFN metabolites might activate Caspase-3 and upregulate Caspase-7 markedly^51^. Similarly, here we found that PTX and SFN metabolites activated Caspase-3 and increased microtubule disruption leading to apoptosis. Furthermore, as the SFN metabolites, SFN-Cys and SFN-NAC have higher enrichment in lung cancer tissue and longer half-life in circulation. Combination of PTX and SFN metabolites not only reduced the working doses of sulforaphane metabolites, but also lowered the resistance to PTX increasing anti-cancer efficiency and apoptosis.

In conclusion, although PTX therapy made an amazing success in cancer treatment, the increasing side-effects and acquired resistance to PTX limited its clinical efficacy. Combination of PTX with SFN metabolites will enhance efficiency via microtubule disruption-caused lower resistance and working doses. Particularly, SFN metabolites can be used for pre-clinical trial since a safe working dose was determined.
